# Early Postoperative Albumin and Neutrophil Dynamics for Risk Stratification After Cytoreductive Surgery in Ovarian Cancer: A Retrospective Multicenter Cohort Study

**DOI:** 10.3390/medicina62030426

**Published:** 2026-02-24

**Authors:** Carlo Ronsini, Antonino Di Nuzzo, Mariano Catello Di Donna, Cono Scaffa, Maria Cristina Solazzo, Stefano Restaino, Martina Arcieri, Giuseppe Vizzielli, Vito Chiantera

**Affiliations:** 1 Unit of Gynecologic Oncology, National Cancer Institute, IRCCS, Fondazione “G. Pascale”, 80131 Naples, Italy; dinuzzo.antonino@gmail.com (A.D.N.); mariano.didonna@istitutotumori.na.it (M.C.D.D.); c.scaffa@istitutotumori.na.it (C.S.); mariacristinasolazzo@gmail.com (M.C.S.); vito.chiantera@istitutotumori.na.it (V.C.); 2 Unit of Gynecology and Obstetrics, Policlinico “G. Martino”, Department of Human Pathology of Adult and Childhood “G. Barresi”, University of Messina, 98100 Messina, Italy; 3 Unit of Obstetrics and Gynecology, “Santa Maria della Misericordia” University Hospital, Azienda Sanitaria Universitaria Friuli Centrale, 33100 Udine, Italy; restaino.stefano@gmail.com (S.R.); martina.arcieri89@gmail.com (M.A.); giuseppevizzielli@yahoo.it (G.V.); 4 Unit of Gynecology and Obstetrics, Department of Woman, Child and General and Specialized Surgery, University of Campania “Luigi Vanvitelli”, 80138 Naples, Italy

**Keywords:** serum albumin, ovarian cancer, postoperative complications, cytoreductive surgery, systemic inflammation

## Abstract

*Background and Objectives*: Serum albumin is a widely available and inexpensive biomarker that reflects nutritional status and physiological reserve. Hypoalbuminemia has been linked to poor postoperative outcomes in surgical oncology; however, its role in predicting early complications after cytoreductive surgery for ovarian cancer, as well as the potential contribution of systemic inflammatory indices in nutritionally preserved patients, remains incompletely understood. This study aimed to evaluate the predictive value of early postoperative serum albumin for early surgical complications and to explore whether inflammatory indices could offer additional prognostic information in patients with adequate albumin levels. *Materials and Methods*: We conducted a retrospective observational cohort study including patients undergoing cytoreductive surgery for ovarian cancer at two Italian tertiary referral centers between July 2023 and December 2025. Postoperative serum albumin was measured on the first postoperative day. Systemic inflammatory parameters were assessed using perioperative changes in neutrophils and composite indices. Early postoperative complications occurring within 30 days were recorded. Multivariable logistic regression analyses were performed, and subgroup analyses were conducted in patients with postoperative albumin ≥3 g/dL. Receiver operating characteristic (ROC) analysis was used to identify an optimal cutoff for significant inflammatory predictors. *Results*: A total of 121 patients were included, of whom 30 developed early postoperative complications. Patients with complications had significantly lower postoperative albumin levels than those without complications (median 2.75 vs. 3.09 g/dL; *p* < 0.001). In multivariable analysis, lower postoperative albumin independently predicted early complications (OR 0.26, 95% CI 0.06–0.86). In the subgroup of patients with preserved albumin levels (≥3 g/dL), a smaller postoperative neutrophil decline independently predicted complications (OR 1.56, 95% CI 1.12–2.70). A neutrophil drop cutoff of −1.15 × 10^3^/dL showed good specificity (81.5%) and high negative predictive value (95.7%). *Conclusions*: Early postoperative serum albumin is a strong predictor of early surgical complications after cytoreductive surgery for ovarian cancer. In patients with preserved nutritional status, dynamic neutrophil changes provide additional prognostic information. Incorporating low-cost metabolic and inflammatory biomarkers may enhance early postoperative risk stratification and support more personalized patient management.

## 1. Introduction

Serum albumin levels are commonly recognized as a dependable marker of nutritional health and overall frailty in patients [1,2]. Although albumin does not capture every aspect of nutritional assessment, it provides a practical overview of protein balance, systemic inflammation, liver function, and metabolic reserve. Significantly, measuring serum albumin is inexpensive, easily accessible, and routinely performed in clinical settings, making it a valuable biomarker for perioperative risk assessment in surgical oncology [3,4]. It is well established that patients with low serum albumin levels are at higher risk of postoperative complications. Hypoalbuminemia has consistently been linked to poor wound healing, increased infectious risk, longer hospital stays, and higher morbidity after various surgical procedures [5]. In gynecologic oncology, especially in ovarian cancer surgery, low albumin levels are associated with adverse perioperative outcomes, likely reflecting pre-existing nutritional deficits and reduced physiological reserve amid the stress of extensive surgery [6,7]. Despite robust evidence supporting the prognostic value of preoperative serum albumin in surgical oncology, the clinical significance of early postoperative albumin as a dynamic marker of surgical stress remains poorly defined. In particular, the predictive value of postoperative day 1 (POD1) serum albumin after cytoreductive surgery for ovarian cancer has not been clearly established. While preoperative albumin reflects baseline nutritional and inflammatory status, early postoperative levels may better capture the immediate physiological impact of extensive surgical debulking, including metabolic stress and acute inflammatory response. In the immediate postoperative period, albumin levels may reflect not only baseline nutritional status but also the acute metabolic response to extensive surgical trauma, systemic inflammation, and perioperative fluid shifts. However, limited data are available on whether early postoperative hypoalbuminemia provides incremental prognostic information for short-term surgical outcomes in patients undergoing cytoreductive surgery for ovarian cancer. Malignant diseases are known to significantly impact systemic inflammatory and immune responses. Tumor-related inflammation can alter white blood cell behavior, cytokine production, and metabolic pathways, fostering a persistent pro-inflammatory state [8]. Recently, composite inflammatory indices derived from routine blood counts have gained attention as diagnostic and prognostic markers in cancer patients [9,10,11,12,13]. These indices are also cost-effective and easy to obtain, as they depend on standard laboratory tests routinely collected in clinical practice. However, it remains unclear whether these inflammatory markers solely reflect tumor biology or also respond to the surgical stress involved. Furthermore, it remains unclear whether perioperative changes in inflammatory parameters provide incremental prognostic information beyond metabolic biomarkers, particularly in patients who maintain adequate early postoperative albumin levels. In such patients, traditional frailty markers may be less discriminatory, and dynamic immune responses may play a more prominent role in determining short-term outcomes. In this context, combining nutritional and inflammatory biomarkers could provide clinicians with a simple, reproducible, and low-cost tool to enhance perioperative risk stratification. Identifying patients at higher risk for postoperative complications through routinely available lab parameters may support more informed clinical decisions and personalized postoperative care. Therefore, this study was designed to address two specific gaps: first, to determine whether POD1 serum albumin independently predicts 30-day postoperative complications after cytoreductive surgery for ovarian cancer; and second, to assess whether dynamic inflammatory indices provide additional prognostic information in patients with preserved postoperative albumin levels.

## 2. Materials and Methods

### 2.1. Study Design

We performed a retrospective observational cohort study including patients who underwent cytoreductive surgery for ovarian cancer at two tertiary referral centers: the Unit of Gynecologic Oncology of the National Cancer Institute, IRCCS, Fondazione “G. Pascale” (Naples, Italy), and the Unit of Obstetrics and Gynecology of the “Santa Maria della Misericordia” University Hospital, Azienda Sanitaria Universitaria Friuli Centrale (Udine, Italy). The study design and reporting were developed in accordance with the Strengthening the Reporting of Observational Studies in Epidemiology (STROBE) guidelines [14]. At both participating university hospitals, all patients provided written informed consent for the anonymous use of clinical data for research purposes. The primary objective was to evaluate the association between postoperative serum albumin levels and the occurrence of early surgical complications. Additionally, a predefined subgroup analysis was conducted among patients with preserved nutritional status, defined by normal albumin levels (≥3 g/dL), to explore whether other systemic inflammatory response markers could independently predict postoperative complications in this population.

### 2.2. Setting

From July 2023 to December 2025, all consecutive patients undergoing cytoreductive surgery for ovarian cancer at the two participating institutions were assessed for postoperative nutritional status by measuring serum albumin levels and body mass index (BMI). Patients were then grouped based on the occurrence of early postoperative surgical complications. Additionally, serum levels of lymphocytes, neutrophils, monocytes, and platelets were measured before and after surgery in all patients to assess the systemic inflammatory response and explore its possible link to postoperative complications. Perioperative and postoperative management protocols, including nutritional assessment and support, fluid management, and postoperative monitoring, were standardized and shared between the participating centers. Both institutions follow comparable clinical pathways for patients undergoing cytoreductive surgery for ovarian cancer, ensuring homogeneity in perioperative care. Perioperative management was standardized across both institutions. All patients received early postoperative nutritional support starting on postoperative day 1 according to institutional protocols. In cases of documented postoperative hypoalbuminemia, albumin supplementation was administered according to best clinical practice after laboratory confirmation of low levels. Importantly, hypoalbuminemia itself did not constitute an indication for ICU transfer or intensified monitoring. No additional diagnostic or therapeutic interventions were performed solely on the basis of albumin values or neutrophil dynamics. Any further interventions (e.g., antibiotics, drainage procedures, reoperation) were undertaken only after the clinical diagnosis of specific postoperative complications.

### 2.3. Participants

Eligibility criteria required the availability of complete clinical and surgical data, including detailed information on cytoreductive procedures, patient age and body mass index (BMI) at the time of treatment, and a confirmed histopathological diagnosis of ovarian cancer. Enrollment further required a complete blood count obtained within 7 days prior to surgery and repeated within 24 h after the procedure. Measurement of serum albumin levels on the first postoperative day was also mandatory for study inclusion. Patients were excluded if they had pre-existing chronic systemic inflammatory or autoimmune conditions, including but not limited to Crohn’s disease, ulcerative colitis, systemic lupus erythematosus, multiple sclerosis, Hashimoto’s thyroiditis, non-alcoholic fatty liver disease, fibromyalgia, chronic kidney disease, hepatitis, osteoarthritis, or psoriasis. Additional exclusion criteria included a histological diagnosis of ovarian or extra-ovarian endometriosis, a history of other malignancies diagnosed within the previous three years, disorders associated with endogenous corticosteroid overproduction, or exposure to systemic steroid therapy within 30 days before blood sampling. Finally, patients with a postoperative follow-up of less than 30 days were excluded from the analysis. Patient identification was performed retrospectively through institutional clinical databases at the two participating centers. All cases meeting the predefined inclusion criteria during the study period were consecutively selected. Because patients were extracted directly from a comprehensive institutional database based strictly on eligibility criteria, no intermediate screening steps were performed, and no additional post hoc exclusions were applied beyond those explicitly defined above.

### 2.4. Variables

A wide range of demographic, surgical, pathological, and laboratory variables was evaluated. Body mass index (BMI, kg/m^2^) and age (years) were analyzed as continuous variables, while ethnicity was treated as a categorical variable. Surgical complexity was quantified using the Aletti Surgical Complexity Score (SCS) [15], analyzed as a continuous measure. The timing of cytoreduction was classified as primary debulking surgery (PDS) or interval debulking surgery (IDS). Tumor stage was assigned according to the FIGO 2014 classification [16], and histological subtype was modeled as an ordinal variable. Homologous recombination status was categorized as proficient or deficient. Postoperative residual disease was classified as CC0, indicating complete macroscopic cytoreduction, CC1 for residual disease ≤1 cm, and CC2 for residual disease >1 cm. Preoperative anesthesiological risk was assessed using the American Society of Anesthesiologists (ASA) score and treated as an ordinal variable. Finally, the usage of HIPEC is a dichotomous variable. Peripheral blood counts, including neutrophils, monocytes, lymphocytes, and platelets, were recorded as continuous variables and expressed as 10^3^ units/dL. Serum albumin concentration was also analyzed as a continuous variable (g/dL). A postoperative serum albumin cutoff of 3.0 g/dL was used to define preserved nutritional status. This threshold reflects the lower limit of the normal reference range routinely adopted by the clinical laboratories of the participating institutions and is commonly used in daily clinical practice to identify clinically relevant hypoalbuminemia. Albumin was analyzed as an absolute early postoperative value rather than as a perioperative change (Δ albumin). This choice was based on prior literature demonstrating a robust association between early postoperative hypoalbuminemia and surgical complications, independently of baseline levels. Conversely, inflammatory indices were evaluated dynamically (Δ values) to better capture the magnitude and direction of the postoperative inflammatory response. These hematological parameters were further integrated into two composite inflammatory indices: the Systemic Inflammatory Response (SIR), defined as neutrophils × platelets lymphocytes, and the Systemic Inflammatory Response Index (SIRI), defined as monocytes × platelets lymphocytes. Both indices were handled as continuous variables in the analyses. Early postoperative complications were defined as the occurrence of any surgical complication classified according to the Clavien–Dindo system within 30 days after surgery, regardless of severity [17]. For statistical purposes, complications were analyzed as a binary variable (presence vs. absence of any Clavien–Dindo grade I–V event within 30 days), given the limited number of severe events. This approach was intentionally adopted to capture the overall burden of postoperative morbidity, including both minor and major events, as even low-grade complications may reflect impaired physiological reserve and negatively influence recovery trajectories after extensive cytoreductive surgery.

### 2.5. Laboratory

Peripheral venous blood samples (3.0 mL) were collected from the antecubital vein at two predetermined time points: within seven days before cytoreductive surgery and again on the first postoperative day. For hematological analyses, blood was drawn using a sterile vacuum collection system into tubes containing ethylenediaminetetraacetic acid (EDTA) as an anticoagulant. Samples were gently inverted immediately after collection and temporarily stored at 4 °C. All specimens were processed within two hours using an automated hematology analyzer to determine absolute counts of neutrophils, lymphocytes, monocytes, eosinophils, basophils, and platelets, with results expressed as 10^3^ units/dL. Serum albumin concentration was measured from blood samples obtained on the first postoperative day. Samples were collected in serum separator tubes, allowed to clot at room temperature, and then centrifuged following standardized laboratory procedures to isolate serum. Albumin levels were quantified using an automated biochemical assay routinely used in clinical diagnostics, with results reported in g/dL. All blood sample processing and laboratory measurements were conducted in-house at both participating institutions, following harmonized protocols and quality control procedures to reduce inter-laboratory variability and ensure accuracy and reproducibility.

### 2.6. Statistical Analysis

The null hypothesis of the study was that postoperative serum albumin levels do not differ between patients who developed early surgical complications and those who did not (H_0_: μ_albumin, complications = μ_albumin, no complications). Continuous variables were initially explored using histograms and boxplots to assess distributional characteristics and identify potential outliers. As most continuous variables showed a non-normal distribution, data were summarized as medians with interquartile ranges (IQRs) and compared between groups (complication yes vs. no) using the Kruskal–Wallis test. Categorical and ordinal variables were reported as absolute frequencies and percentages and compared using Fisher’s exact test. Multivariable logistic regression models were constructed to evaluate the association between selected clinical, surgical, and laboratory variables and the occurrence of early postoperative complications. Variables entered into the multivariable model were selected based on statistical significance in univariable analysis. This approach was adopted to limit the number of covariates relative to the number of events and to reduce the risk of model overfitting, given the sample size and event rate. Clinically relevant variables such as ASA score and disease stage were evaluated in univariable analyses but were not retained in the final model if not statistically associated with the outcome. Effect estimates were reported as odds ratios (ORs) with corresponding 95% confidence intervals (CIs), and model significance was assessed using likelihood-based methods. All analyses were subsequently repeated in a predefined subgroup of patients with normal postoperative albumin levels (≥3 g/dL), in order to investigate whether, among individuals with preserved nutritional status, systemic inflammatory response parameters could predict early postoperative complications. For the inflammatory index that remained significantly associated with complications in this subgroup, receiver operating characteristic (ROC) curve analysis was performed, and the optimal cutoff value was determined using the Youden index. The primary objective of the regression analysis was to assess independent associations between early postoperative biomarkers and 30-day complications rather than to develop or validate a predictive model. Therefore, model discrimination metrics (e.g., c-statistic/AUC) were not formally evaluated.

An ex post power analysis was conducted based on the observed difference in postoperative albumin levels between groups and the final study sample size (n = 121). Assuming a two-sided alpha level of 0.05, the achieved statistical power was greater than 80%, indicating that the study was adequately powered to detect clinically meaningful differences in the primary outcome.

All statistical tests were two-sided, and a *p*-value < 0.05 was considered statistically significant. Statistical analyses were performed using R software and RStudio (version 2023.12.1+402).

### 2.7. Risk of Bias

Multivariable logistic regression analyses were conducted to identify independent predictors of early postoperative complications while controlling for potential confounders. Consistent with the predefined analytical plan, only variables demonstrating a statistically significant association with the outcome in univariable analysis were included in the multivariable models. Competing models were evaluated using the Bayesian Information Criterion (BIC) and adjusted goodness-of-fit measures, with the most parsimonious model selected based on the lowest BIC value, as previously described. Given the limited number of events, model complexity was intentionally restricted to avoid overfitting. All statistical analyses were independently performed by two investigators (CR and ADN). The second analysis was carried out under blinded conditions, with the investigator unaware of the study objectives, to minimize analytical bias. No missing data were observed for the outcomes of interest.

## 3. Results

### 3.1. Study Population and Baseline Characteristics

A total of 121 patients undergoing cytoreductive surgery for ovarian cancer were included in the final analysis. Early postoperative surgical complications occurred in 30 patients, while 91 patients did not develop complications within 30 days after surgery. Baseline demographic, clinical, surgical, and pathological characteristics stratified by complication status are summarized in Table 1.

Patients who developed postoperative complications had a significantly higher body mass index compared with those without complications (median BMI 27.6 vs. 23.6 kg/m^2^, *p* < 0.001) and underwent more complex surgical procedures, as reflected by a higher Surgical Complexity Score (median SCS 18 vs. 9, *p* < 0.001). No statistically significant differences were observed between the two groups in terms of age, ethnicity, menopausal status, FIGO stage distribution, histological subtype, homologous recombination status (*p* = 0.40), type of surgery, residual disease, ASA score, or use of HIPEC.

### 3.2. Outcomes

**Figure 1:**
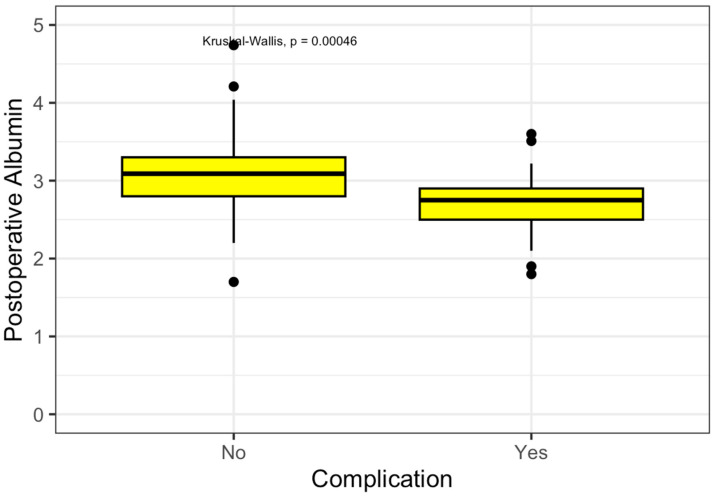
Postoperative Albumin’s levels.

The primary outcome of the study was postoperative serum albumin concentration. Patients who developed early postoperative complications had significantly lower postoperative albumin levels than those who did not (median 2.75 g/dL [IQR 0.40] vs. 3.09 g/dL [IQR 0.50]; *p* < 0.001), as shown in Figure 1.

In contrast, no statistically significant differences were observed between groups in postoperative changes in systemic inflammatory indices. Specifically, the postoperative variation in SIR was comparable between patients with and without complications (median −1003 [IQR 2203] vs. −984 [IQR 1583], *p* > 0.90), as was the postoperative variation in SIRI (median −4.0 [IQR 7.1] vs. −4.3 [IQR 5.4], *p* > 0.90). Similarly, the postoperative neutrophil drop did not differ significantly between the two groups (median −3.5 [IQR 7.3] vs. −4.0 [IQR 5.1] × 10^3^/dL, *p* = 0.50). Those data are shown in Table 2.

### 3.3. Multivariable Analysis of Postoperative Albumin and Complications

Multivariable logistic regression analysis, including variables that were significantly different at univariable comparison, confirmed postoperative serum albumin as an independent predictor of early surgical complications. Lower postoperative albumin levels were associated with an increased risk of complications (OR 0.26, 95% CI 0.06–0.86, *p* = 0.041). Higher BMI (OR 1.20, 95% CI 1.08–1.36, *p* = 0.002) and higher surgical complexity (OR 1.17, 95% CI 1.08–1.28, *p* < 0.001) also remained independently associated with the development of postoperative complications (Table 3).

### 3.4. Subgroup Analysis in Patients with Preserved Albumin Levels

**Figure 2:**
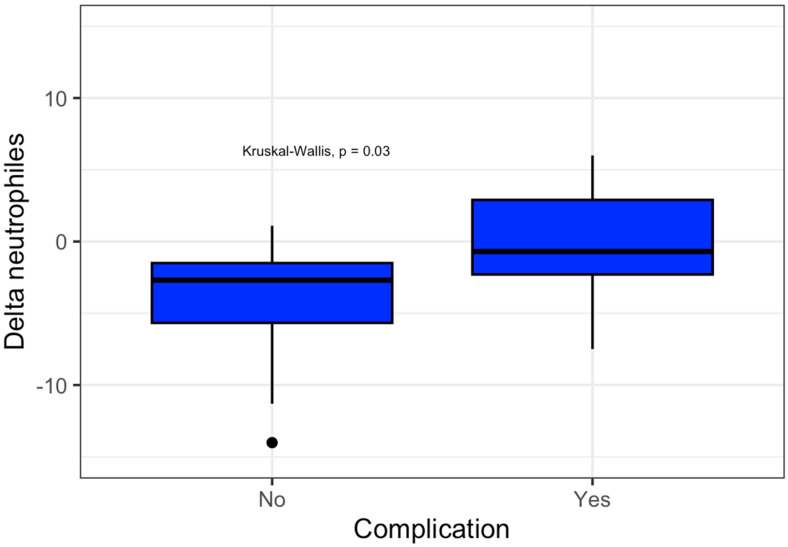
Δ Neutrophils in patients with albumin levels ≥3 g/dL.

A predefined subgroup analysis was performed in patients with normal postoperative albumin levels (≥3 g/dL). Within this population, patients who developed complications showed a significantly smaller postoperative decline in neutrophil count compared with those without complications (median Δ neutrophils −0.7 vs. −2.7 × 10^3^/dL, *p* = 0.031) (Figure 2).

Surgical complexity was also significantly higher in patients with complications (median SCS 21 vs. 8, *p* = 0.036). No significant differences were observed for ΔSIR (median −212 vs. −880, *p* = 0.30), ΔSIRI (median −0.7 vs. −3.4, *p* = 0.20), or BMI (median 27.3 vs. 23.4 kg/m^2^, *p* = 0.12) (Table 4).

In multivariable logistic regression analysis restricted to patients with postoperative albumin ≥3 g/dL, a smaller postoperative neutrophil decrease remained independently associated with early complications (OR 1.56, 95% CI 1.12–2.70, *p* = 0.045), together with higher surgical complexity (OR 1.22, 95% CI 1.06–1.49, *p* = 0.020) (Table 5).

### 3.5. ROC Analysis and Cutoff Determination for Neutrophil Variation

**Figure 3:**
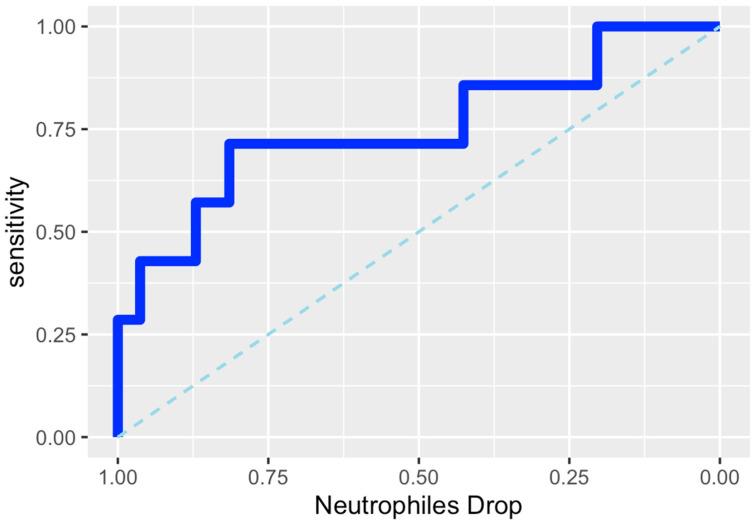
ROC Curve for Δ Neutrophils −1.15.

Receiver operating characteristic (ROC) analysis identified a postoperative neutrophil drop of −1.15 × 10^3^/dL as the optimal cutoff for predicting early complications in patients with preserved albumin levels. At this threshold, sensitivity was 71.4% (95% CI 29.0–96.3) and specificity was 81.5% (95% CI 68.6–90.7). The positive predictive value was 33.3% (95% CI 11.8–61.6), while the negative predictive value was 95.7% (95% CI 85.2–99.5). The positive likelihood ratio was 3.86 (95% CI 1.86–8.00) and the negative likelihood ratio was 0.35 (95% CI 0.11–1.14), corresponding to an odds ratio of 11.0 (95% CI 1.86–65.08). Overall accuracy was 80.3% (95% CI 68.2–89.4), with an error rate of 19.7%. The ROC curve is shown in Figure 3.

## 4. Discussion

### 4.1. Interpretation of Results

The present study shows that early postoperative serum albumin is the strongest predictor of early surgical complications after cytoreductive surgery for ovarian cancer. Patients who experienced complications had significantly lower postoperative albumin levels, whereas no notable differences were observed in overall postoperative inflammatory markers across the entire group. These results support the idea that postoperative hypoalbuminemia is not just a biochemical abnormality, but also a marker of reduced physiological reserve and increased vulnerability to surgical stress. Importantly, surgical burden is a major determinant of early postoperative physiology. In our cohort, the Surgical Complexity Score (SCS) was markedly higher among patients who developed complications and remained independently associated with the outcome in multivariable models. Extensive cytoreductive procedures may contribute to early hypoalbuminemia through greater surgical trauma, capillary leak, hemodilution from perioperative fluid administration, and increased protein catabolism; likewise, early leukocyte kinetics may be influenced by the magnitude of tissue injury and the intensity of the postoperative inflammatory response. Although we adjusted for SCS in the multivariable analyses, we acknowledge that residual confounding related to operative extent (e.g., blood loss, operative time, transfusions, fluid balance, and unmeasured components of surgical stress) cannot be fully excluded and may partially account for the observed associations. Therefore, our findings should be interpreted as risk stratification signals rather than evidence of causality. The emphasis on postoperative day 1 measurements reflects the hypothesis that the early systemic response to surgical stress may reveal individual vulnerability before overt clinical deterioration occurs. While serial measurements could further refine mechanistic understanding, the clinical value of POD1 biomarkers lies in their potential to identify high-risk patients at a stage when corrective strategies remain feasible. Patients with hypoalbuminemia are often in a weakened state, which includes malnutrition, chronic inflammation, sarcopenia, and decreased liver synthetic function [18,19]. In this context, lower postoperative albumin levels likely indicate both pre-existing frailty and an inadequate metabolic response to the stress of extensive cytoreductive surgery. Albumin plays key roles in maintaining blood oncotic pressure, regulating inflammatory responses, transporting molecules, and buffering oxidative stress [20,21]. A drop in albumin levels may therefore increase the risk of poor wound healing, infections, and a decreased ability to handle perioperative stress, leading to a higher chance of early postoperative complications. Albumin should be interpreted as a surrogate marker rather than a direct therapeutic target. Conversely, when postoperative albumin levels are maintained, indicating good nutritional and metabolic health, the factors predicting postoperative problems change. In this group, static inflammatory markers become less informative, whereas dynamic immune parameters, especially the postoperative change in neutrophil count, stand out as independent predictors of complications. The unique prognostic importance of neutrophil dynamics, compared to combined inflammatory indices such as SIR and SIRI, may be due to the biological specificity and timing sensitivity of neutrophils in surgical stress. Neutrophils are the first line of defense in innate immunity and quickly respond to tissue injury, ischemia–reperfusion damage, and microbial invasion [22,23,24]. Their perioperative behavior thus closely reflects the body’s immediate response to surgical trauma. In contrast, indices like SIR and SIRI combine multiple immune cell types and platelet counts into ratios, which might dilute short-term, cell-specific signals. While these indices effectively reflect chronic or systemic inflammation, they may be less responsive to the acute immune changes after surgery, especially in patients with good nutritional and metabolic reserves. In such cases, normal inflammatory balance may lessen the usefulness of overall indices, while small changes in neutrophil activity, activation, or removal could still indicate clinically important immune dysregulation. This supports the idea that in patients with adequate nutrition, postoperative complications are less driven by overall frailty and more influenced by how the immune system responds to surgery. A less pronounced decrease in neutrophil count may indicate ongoing immune activation, poor inflammation resolution, or early, hidden complications, all of which could become clinically significant before obvious symptoms develop. Importantly, this subgroup analysis was based on a limited number of patients and events, which increases the risk of overfitting and imprecise effect estimation. The wide confidence intervals around the ROC-derived cutoff underscore the need for external validation in larger cohorts before any clinical application.

### 4.2. Comparison with Existing Literature

The link between hypoalbuminemia and adverse postoperative outcomes has been widely documented across various surgical fields [1,2]. Numerous studies indicate that both preoperative and postoperative albumin levels are strong predictors of complications, death, and hospital stay duration in cancer surgeries [3,4,5]. In gynecologic oncology, low albumin levels have been linked to higher postoperative complications and reduced survival in ovarian cancer patients undergoing cytoreductive surgery [6,7]. Our findings support this body of evidence and highlight the importance of early postoperative albumin measurement as a practical, useful biomarker. Conversely, the prognostic value of systemic inflammatory indices, such as SIR and SIRI, in the perioperative period remains unclear. While these markers have been associated with long-term cancer outcomes and survival in ovarian and other cancers, their ability to forecast short-term surgical complications remains inconsistent. Our results align with studies suggesting that composite inflammatory scores may be insufficiently sensitive during the immediate postoperative phase when evaluated in broad populations. Importantly, the subgroup-specific significance of neutrophil behavior observed in our study aligns with emerging evidence that immune responses, rather than absolute numbers, are more meaningful. Past research has indicated that abnormal perioperative neutrophil responses are linked to postoperative infections, slower recovery, and weakened immune defense [25,26]. Our data expand on these findings by showing that, among patients with adequate nutritional status, a limited drop in neutrophil count after surgery identifies those at higher risk for early complications, supporting the idea that a maladaptive or prolonged inflammatory response can be detrimental.

### 4.3. Clinical Implication

From a clinical perspective, these findings endorse routine measurement of serum albumin on the first postoperative day as a key part of early postoperative risk assessment after cytoreductive surgery. Due to its low cost, widespread availability, and strong prognostic significance, postoperative albumin could serve as an early warning sign to identify patients needing increased surveillance, early nutritional support, or proactive management of potential complications. In this context, early postoperative hypoalbuminemia might be a modifiable risk factor. Although causality cannot be established in this study, early detection of low albumin levels could facilitate the timely implementation of targeted nutritional interventions. In addition to optimized enteral or parenteral nutrition, early albumin supplementation may be considered for selected patients with significant postoperative hypoalbuminemia and a high surgical burden. Albumin replacement has been shown to restore oncotic pressure, enhance intravascular volume distribution, and exert immunomodulatory and antioxidant effects, especially in critically ill and surgical patients [27,28]. From a clinical perspective, early postoperative albumin measurement may serve as a simple tool for risk stratification rather than as a direct therapeutic target. Although hypoalbuminemia has been associated with adverse surgical outcomes, our observational design does not allow causal inferences regarding the potential benefit of albumin supplementation. Therefore, our findings should not be interpreted as supporting corrective albumin administration as an evidence-based intervention. Instead, early hypoalbuminemia may function as a marker of physiological stress and vulnerability, identifying patients who could benefit from closer monitoring and optimized perioperative management. Whether targeted albumin replacement improves outcomes in this setting requires a prospective randomized investigation. At present, albumin should be considered a prognostic biomarker rather than a modifiable therapeutic determinant in this context. Additionally, assessing postoperative neutrophil dynamics in patients with normal albumin levels provides a complementary, more detailed risk-stratification approach. The neutrophil drop cutoff identified demonstrated good specificity and a high negative predictive value, indicating its potential usefulness in identifying low-risk patients who can safely follow standard postoperative pathways. Given the exploratory nature of subgroup analyses, results should be interpreted cautiously. Although exploratory, this method underscores the potential of combining early metabolic optimization with dynamic immune monitoring to customize postoperative care, especially in high-risk surgical oncology cases.

### 4.4. Strengths and Limitations

The strengths of this study include its focus on early postoperative biomarkers, the use of dynamic inflammatory parameters, and predefined subgroup analyses based on nutritional status, all of which enhance biological plausibility and clinical relevance. The standardized surgical approach and perioperative management across two referral centers further strengthen the internal validity of the findings. However, several limitations must be acknowledged. The retrospective design restricts causal inference and may be affected by unmeasured confounding despite multivariable adjustment. In particular, although surgical complexity (Aletti SCS) was included in the models, unmeasured features of operative burden and perioperative management (e.g., estimated blood loss, transfusions, operative time, fluid balance) may still confound the relationship between early postoperative biomarkers (albumin and neutrophil dynamics) and complications. These strict exclusion criteria were adopted to minimize confounding on inflammatory markers but may limit external validity. The relatively small sample size, especially within the subgroup of patients with preserved postoperative albumin levels, reduces statistical power and warrants caution when interpreting cutoff-based analyses. Additionally, the study population was exclusively derived from two Italian referral centers, which may limit the generalizability of the results to other geographic or healthcare settings with different patient characteristics, surgical practices, or perioperative management protocols. Although this was a multicenter study, standardized perioperative pathways likely mitigated center-related variability. Another significant limitation is the absence of stratification based on the severity of postoperative complications. By categorizing complications as a binary outcome (present vs. absent), the analysis may not fully capture the clinical heterogeneity or true burden of postoperative morbidity. The primary outcome was defined as the presence of any postoperative complication, without stratification by Clavien–Dindo severity. While this approach may introduce heterogeneity by combining minor and major events, it was chosen to avoid underestimating the overall postoperative morbidity and to enhance the sensitivity of early risk stratification. Furthermore, complications were analyzed as a composite binary endpoint including all Clavien–Dindo grades (I–V). While this approach maximizes statistical power in relatively small cohorts, it may dilute associations by combining minor and major events and potentially obscure severity-specific gradients. The limited number of high-grade complications in our sample precluded adequately powered severity-stratified sensitivity analyses (e.g., ≥Grade II or ≥Grade III). Therefore, our findings should be interpreted as reflecting overall postoperative morbidity rather than predictors of major complications specifically. Future larger, prospective studies should evaluate whether early postoperative albumin and leukocyte dynamics differentially predict clinically relevant (≥Grade III) morbidity. Nevertheless, future studies with larger sample sizes should investigate whether the observed associations differ according to complication severity. This approach could obscure differences between minor and major complications, potentially weakening or hiding the strength of the observed associations. Although covariate selection was guided by univariable significance to preserve model stability, this data-driven approach may have excluded clinically relevant factors not reaching statistical significance, potentially resulting in residual confounding. Finally, inflammatory markers were measured at predefined perioperative time points and may not reflect the full complexity or temporal progression of postoperative immune responses. Another important limitation is that albumin and inflammatory parameters were measured at predefined early perioperative time points, with postoperative assessment performed on day 1 only. Serial longitudinal measurements were not available for the present analysis. Therefore, we cannot characterize the full temporal trajectory of postoperative metabolic and immune responses, nor assess whether persistent or delayed alterations would demonstrate stronger associations with complications. However, the rationale of this study was to investigate whether very early postoperative changes, reflecting the immediate physiological response to surgical trauma, could serve as practical and actionable risk-stratification markers. Later or prolonged laboratory deterioration may indeed reflect evolving complications but would provide less opportunity for timely preventive intervention. Accordingly, our findings should be interpreted as prognostic associations based on early postoperative physiology rather than evidence of causal relationships. External validation in independent cohorts and prospective studies, ideally including graded complication severity, is necessary before these findings can be applied in routine clinical practice.

## 5. Conclusions

Early postoperative serum albumin is a simple and reliable biomarker for identifying patients at increased risk of early surgical complications after cytoreductive surgery for ovarian cancer. Postoperative hypoalbuminemia indicates reduced physiological reserve and vulnerability to surgical stress, while dynamic immune parameters, especially postoperative neutrophil variation, offer additional prognostic information in patients with normal albumin levels. Combining early metabolic and inflammatory biomarkers may enhance postoperative risk assessment and support more personalized patient management. Further prospective studies are needed to validate these findings and clarify their potential clinical impact.

## Figures and Tables

**Table 1:** Patients’ Characteristics.

Characteristic	No, N = 91 ^1^	Yes, N = 30 ^1^	*p*-Value ^2^
Age	64, (19)	61, (20)	0.3
BMI	23.6, (5.8)	27.6, (4.4)	<0.001
Ethnicity			0.4
Caucasian	90, (99%)	29, (97%)	
Hispanic	1, (1.1%)	0, (0%)	
Middle East	0, (0%)	1, (3.3%)	
Menopause	78, (86%)	25, (83%)	0.8
HR			0.4
Deficient	32, (52%)	7, (47%)	
Proficient	29, (47%)	7, (47%)	
Missing	30	16	
Histotype			0.8
Clear Cell	2, (2.2%)	1, (3.3%)	
Endometrioid	2, (2.2%)	1, (3.3%)	
HGSOC	78, (86%)	25, (83%)	
LGSOC	4, (4.4%)	2, (6.7%)	
Mucinous Expensive	3, (3.3%)	0, (0%)	
Mucinous Infiltrative	2, (2.2%)	1, (3.3%)	
FIGO stage			0.14
II	4, (4.4%)	0, (0%)	
III	61, (67%)	16, (53%)	
IV	26, (29%)	14, (47%)	
Surgery Type			0.2
IDS	49, (54%)	12, (40%)	
PDS	42, (46%)	18, (60%)	
SCS	9, (10)	18, (7)	<0.001
CC			0.3
0	77, (86%)	25, (83%)	
1	8, (8.9%)	5, (17%)	
2	5, (5.6%)	0, (0%)	
Missing	1	0	
ASA			0.11
1	0, (0%)	1, (3.3%)	
2	49, (55%)	14, (47%)	
3	39, (44%)	13, (43%)	
4	1, (1.1%)	2, (6.7%)	
Missing	2	0	
HIPEC	15, (16%)	3, (10%)	0.6

^1^ Median, (IQR); n, (%); ^2^ Wilcoxon rank sum test; Fisher’s exact test; Pearson’s Chi-squared test.

**Table 2:** Outcomes.

Characteristic	No, N = 91 ^1^	Yes, N = 30 ^1^	*p*-Value ^2^
Postoperative Albumin	3.09, (0.50)	2.75, (0.40)	<0.001
Δ SIR	−984, (1583)	−1003, (2203)	>0.9
Δ SIRI	−4.3, (5.4)	−4.0, (7.1)	>0.9
Δ Neutrophils	−4.0, (5.1)	−3.5, (7.3)	0.5

^1^ Median, (IQR). ^2^ Wilcoxon rank sum test.

**Table 3:** Multivariate logistic Regression.

Variable	Estimate	Std. Error	z Value	*p*-Value	Odds Ratio	OR 95% CI
Albumin	−1.365	0.666	−2.049	0.041	0.256	0.062–0.858
BMI	0.182	0.058	3.133	0.002	1.200	1.076–1.355
SCS	0.153	0.042	3.670	0.000	1.165	1.08–1.275

**Table 4:** Subanalysis’ Outcomes.

Characteristic	No, N = 54 ^1^	Yes, N = 7 ^1^	*p*-Value ^2^
Δ SIR	−880, (1468)	−212, (1719)	0.3
Δ SIRI	−3.4, (5.0)	−0.7, (3.1)	0.2
Δ Neutrophils	−2.7, (4.2)	−0.7, (5.2)	0.031
BMI	23.4, (5.8)	27.3, (3.8)	0.12
SCS	8, (12)	21, (9)	0.036

^1^ Median, (IQR). ^2^ Wilcoxon rank sum test.

**Table 5:** Logit Regression Multivariate in patients with Albumin ≥3 g/dL.

Variable	Estimate	Std. Error	z Value	*p*-Value	Odds Ratio	OR 95% CI
Δ Neutrophils	0.442	0.221	2.003	0.045	1.556	1.116–2.697
SCS	0.201	0.086	2.332	0.020	1.223	1.055–1.493

## Data Availability

All data and the methodological process for their calculation have been deposited on Zenodo at https://doi.org/10.5281/zenodo.18436365 (accessed on 7 February 2026). Their use is public.

## Abbreviations

The following abbreviations are used in this manuscript:

ASA	American Society of Anesthesiologists
BIC	Bayesian Information Criterion
BMI	Body Mass Index
CC	Completeness of Cytoreduction
CI	Confidence Interval
EDTA	Ethylenediaminetetraacetic Acid
FIGO	International Federation of Gynecology and Obstetrics
HIPEC	Hyperthermic Intraperitoneal Chemotherapy
IDS	Interval Debulking Surgery
IQR	Interquartile Range
IRB	Institutional Review Board
OR	Odds Ratio
PDS	Primary Debulking Surgery
ROC	Receiver Operating Characteristic
SCS	Surgical Complexity Score
SIR	Systemic Inflammatory Response
SIRI	Systemic Inflammatory Response Index
STROBE	Strengthening the Reporting of Observational Studies in Epidemiology
